# Inverted Conformation Stability of a Motor Molecule on a Metal Surface

**DOI:** 10.1021/acs.jpcc.2c00406

**Published:** 2022-05-18

**Authors:** Monika Schied, Deborah Prezzi, Dongdong Liu, Peter Jacobson, Stefano Corni, James M. Tour, Leonhard Grill

**Affiliations:** † Department of Physical Chemistry, University of Graz, Heinrichstraße 28, 8010 Graz, Austria; ‡ Nanoscience Institute of the National Research Council (CNR-NANO), via G. Campi 213/a, 41125 Modena, Italy; § Departments of Chemistry and Materials Science and NanoEngineering, the Smalley Institute for Nanoscale Science and Technology, the Welch Institute for Advanced Materials, Rice University, Houston, Texas 77005, United States; ∥ Dipartimento di Scienze Chimiche, Università di Padova, Padova I-35131, Italy

## Abstract

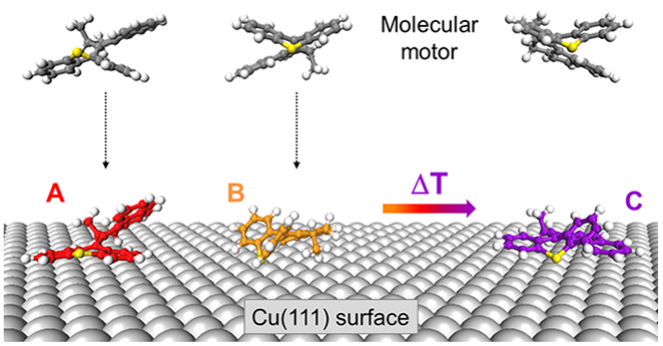

Molecular motors have been intensely studied in solution, but less commonly on solid surfaces that offer fixed points of reference for their motion and allow high-resolution single-molecule imaging by scanning probe microscopy. Surface adsorption of molecules can also alter the potential energy surface and consequently preferred intramolecular conformations, but it is unknown how this affects motor molecules. Here, we show how the different conformations of motor molecules are modified by surface adsorption using a combination of scanning tunneling microscopy and density functional theory. These results demonstrate how the contact of a motor molecule with a solid can affect the energetics of the molecular conformations.

## Introduction

**Figure 1:**
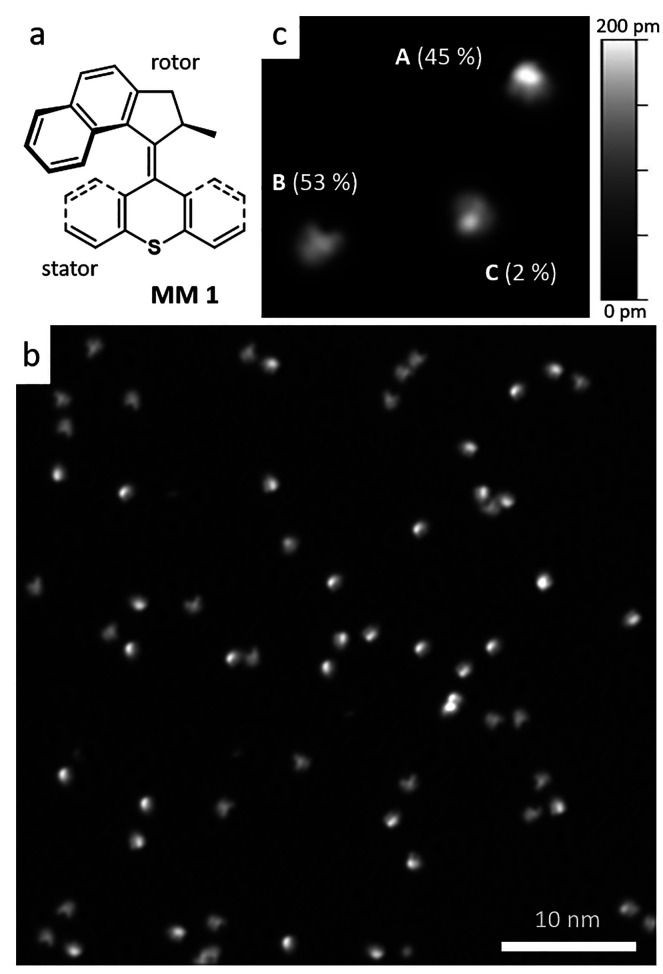
(a) Chemical structure of the **MM1**. (b) STM image (8 pA, 400 mV) of motor molecules on a terrace of the Cu(111) surface. The bright lobes are individual MM1, while the dark spots are CO molecules. (c) Zoom-in STM image (10 pA, 200 mV) showing the three molecular conformations (named A, B, and C with their relative abundance indicated). Deposition was done at 5 K sample temperature.

Molecular motors have attracted much attention in the last two decades, due to their ability to transform energy from an external source into useful motion, for instance, a translation or rotation in one direction. While molecular motors are known in nature,^1,2^ artificial motors have also been designed and successfully synthesized and characterized.^3−5^ An important class of these motors, developed by Feringa and co-workers,^6,7^ is based on a four-step rotary cycle of a chiral, sterically overcrowded alkene. The rotation consists of alternating photoisomerization and thermal isomerization steps. The photochemical step involves an energetically uphill cis–trans isomerization step that causes the molecule to invert helicity before an energetically downhill thermal process, known as the thermal helix inversion, then occurs. The kinetics of the relaxation depend strongly on the steric hindrance between the rotor and stator portion of the molecule (Figure 1a). This steric effect can therefore be modified via side groups, which consequently alters the rotational frequency of the motor.^8^ In the same regard, the total energies of the molecule in its different states play a role. Understanding the conformations of the individual states along the rotation process is therefore crucial for optimization of the motor function. Applying a combination of optical experiments and density functional theory (DFT) calculations, it has been shown that, for molecular motors in solution, small differences in the molecular conformation can affect the motor rotation.^9^

In general, the adsorption of molecules on surfaces is known to modify potential energy surfaces and consequently molecular functions, as has been shown, for instance, in molecular gears^10^ and molecular switches, based on isomerization processes^11^ or intramolecular proton transfer.^12^ Thus, for the case of the more complex Feringa-type molecular motors, it is of great interest whether and how surface adsorption can alter their properties by modifying the different conformations. Here, we show that adsorption of molecular motors at a surface can change these energies. A combination of single-molecule experiments and DFT calculations reveal how the two molecular conformations before and after the helix inversion step are inverted in their thermodynamic stabilities.

Synthetic molecular machines have been intensely studied in solution^13−16^ but less commonly at well-defined surfaces,^17−20^ despite various advantages for surfaces. First, the surface represents a reference point for motion, in particular when following single movements of individual molecular trajectories, making the observations statistically distinct. Second, an isolated motor cannot do useful work as any directional motion is compensated by the conservation of angular momentum—useful work is only made possible when the molecule is influenced by a fixed solid. Moreover, it is the presence of a solid that allows use of scanning tunneling microscopy (STM) and thus imaging with high spatial resolution at the atomic level. Single-crystal surfaces of noble metals are preferred, due to their highly defined periodic surface lattice and very low contamination under ultrahigh vacuum. Here, we have chosen Cu(111) as the substrate, which has already been used for various molecular motors in the last years.^17−19^

The adsorption of functional molecules on a metallic surface can lead to different conformations as compared to the gas phase, due to the molecule–surface interaction. This interaction depends on the molecular structure and is specific for different components within the molecular skeleton. Aromatic ring systems tend to be adsorbed flat on metal surfaces to maximize their interaction with the surface.^21,22^ Experimentally, this has been observed, for example, for azobenzene molecules with the phenyl rings being attracted by the surface,^23^ such as in the so-called Lander molecules where the attraction of a polyaromatic board by the metal surface results in a distortion of sideways attached spacer groups.^24^ The Feringa motor, that we study here, contains aromatic rings and thus one can expect that the conformation of the molecule deforms upon adsorption on a metal and therefore differs from that in solution experiments.

## Methods

Experiments were performed under ultrahigh vacuum (base pressure of ∼2 × 10^–10^ mbar) with a low temperature scanning tunneling microscope (Createc) working at 5 K sample temperature. Imaging is done in the constant-current mode. Cu(111), which has already been used for various molecular motors in the last years,^17−19^ has been chosen as the substrate. After cleaning the metal sample with sputtering and annealing cycles, molecules were deposited from a Knudsen cell onto the clean surface, kept at different temperatures between 5 K and room temperature. For deposition with a constant molecular flux of about 1/2 monolayer (ML) per minute [1 ML is defined as one complete layer of molecules on the Cu(111) surface] and a typical surface coverage of about 0.1 ML, the Knudsen cell was always heated to the same temperature (about 375 K with ∼0.22 W at the filament). After preparation, the sample was transferred into the STM chamber. Deposition of molecules at the lowest temperature was performed from a Si wafer (heated by ∼10 V and 50–100 mA for ∼5 s) directly onto the sample in the STM stage (with a sample temperature of 5 K).

Ab-initio DFT simulations were carried out to investigate the molecular adsorption on Cu(111) by using the Quantum ESPRESSO (QE) package,^25,26^ which features a plane-wave, pseudopotential implementation of DFT. Dispersion forces were included by using an optimized vdW-DF-like^27^ non-local exchange–correlation functional, where the GGA functional is replaced by an optimized Becke86b functional (optB86b-vdW),^28^ which demonstrated to perform extremely well for the description of benzene adsorption on Cu(111).^29^ Ultra-soft pseudopotentials were employed as available in the SSSP library.^30^ The kinetic energy cutoff for the Kohn–Sham wave functions (charge density) was set to 35 (400) Ry. The molecule–surface system was modeled by using an orthorhombic supercell corresponding to a 8 × 4 repetition of a 4-layer slab of Cu(111) (optimized lattice parameter for bulk Cu: *a* = 3.59 Å). A vacuum region of at least 13 Å in the non-periodic direction (i.e., perpendicular to the slab) was introduced to prevent interaction between periodic images, while the in-plane irreducible Brillouin zone was sampled with a 3 × 3 Monkhorst-Pack grid of *k*-points. The ground state configurations were reached through a standard total-energy-and-forces optimization, as implemented in the pw.x code of QE. The atomic positions within the cell were fully relaxed until forces were less than 10 meV/Å. STM images of the optimized configurations were simulated within the Tersoff–Hamann approximation,^31^ where the STM contrast is proportional to the local density of states evaluated at the position of a spherically symmetric (s-wave) tip.

## Results and Discussion

Figure 1a shows the chemical structure of the studied 9-(2′-methyl-2′,3′-dihydro-1′*H*-cyclopenta[*a*]naphthalen-1′-ylidene)-9*H*-thioxanthene molecule (named **MM1** for molecular motor in the following). It follows the design of the Feringa motors,^6,8^ consisting of a stator and a rotor unit (lower and upper part of the molecule in Figure 1a, respectively, as labeled). For a detailed insight into the motor unit, it is smaller and essentially reduced to the motor unit itself without side groups, as compared to other molecules with such a motor unit that have been studied in the past on metallic single-crystal surfaces.^17−19^ The lone methyl group is essential to ensure uni-directionality of rotation by generating diastereomeric transition states. In solution, these molecules are reported to rotate uni-directionally at ∼3 MHz upon light activation.^8^

After deposition of these molecules onto the cold Cu(111) surface (kept at 5 K), individual molecules can be observed on the flat terraces (Figure 1b) in various orientations. Due to the miniscule thermal energy under the preparation conditions, no molecular islands or assemblies are found and the molecules probably adsorb in a hit-and-stick manner. It can be seen already in the large-scale STM image that the molecules appear with varying brightness, that is apparent heights, and shapes. A zoom-in into a smaller area, together with a statistical analysis of many molecules in larger areas, reveals that **MM1** adsorbs in three appearances, which are called A, B, and C (Figure 1c). They all have approximately the same size, but differ in their precise shape. A and B are the most abundant ones (45 and 53% of the molecules, respectively), while C is very rare (only 2%). We have used rather low bias voltages for stable imaging of the molecules since higher bias voltages can induce rotation or translation of the molecules.^32^

**Figure 2:**
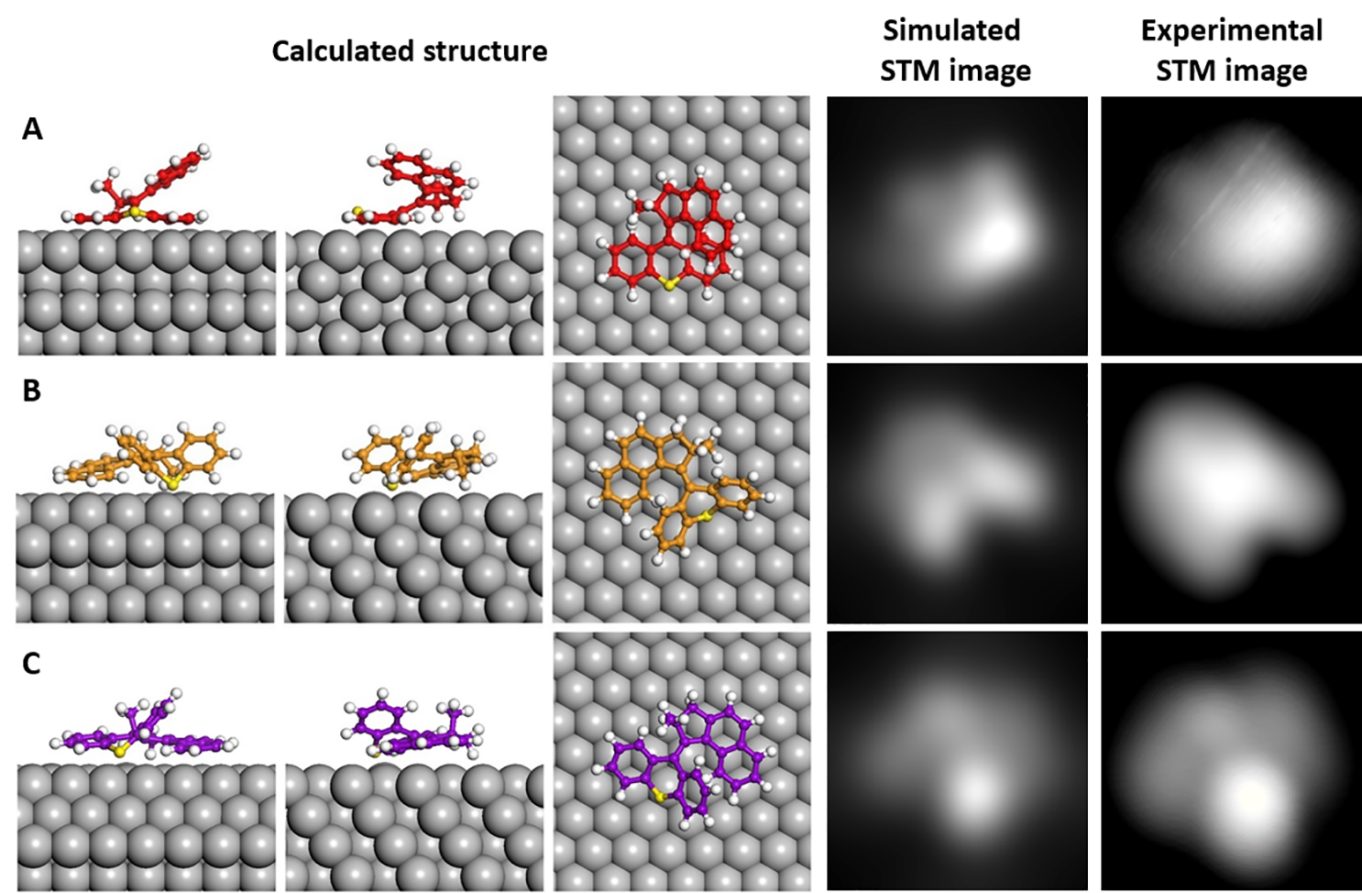
Comparison of the calculated structures on the Cu(111) surface (at the left: two side views and one top view) with simulated and experimental STM images [all 1.8 nm × 1.8 nm in size with 9 pA/200 mV (A), 10 pA/100 mV (B) and 60 pA/−50 mV (C)] for all three molecular motor conformations (A, B, and C) as indicated. All structures and images show the (S) enantiomer (see Figure 3 below). The simulated STM images were artificially blurred (see Figure S1).

The observation of three molecular appearances on the surfaces suggests the presence of different molecular conformations, potentially related to the configurations that the molecular motor can adapt in solution during a rotation cycle. In order to get atomistic insights, we have performed first principles DFT-based simulations by considering two main starting configurations, with **MM1** having either the stator or the rotor (see Figure 1a) facing the surface. In addition, we considered for the latter also the case of the less stable isomer, with the methyl group pointing upward [(P)-1 state of the rotation cycle; as discussed below]. In this configuration, the aromatic part of the rotor is exposed more strongly to the surface. From these optimized structures (at the left of Figure 2), we have simulated STM images and compared them to the experimental images (at the right of Figure 2), finding very good agreement.

With the knowledge of the adsorption structures on the surface and their DFT energetics, one can now analyze the meaning of these conformations. First, we notice that the sulfur atom in the stator can mostly interact with the Cu(111) surface when the molecule lands in rotor-down configuration on the surface, that is B and C conformations, where the closest S–Cu distance is ∼2.4 Å, while it is less relevant in A (S–Cu distance >3.4 Å). Second, we find that in all three cases, the aromatic groups tend to lie flat on the surface, a typical behavior on metal surfaces where the delicate interplay between van der Waals and π-electron interactions determines the actual registry on the metallic surface and the strength of the adsorption.^21−23^ For conformation A, the stator flattens on the surface and tends to lie in registry with the hcp hollow site (top in Figure 2), in agreement with the lowest energy configuration of other aromatic molecules on Cu(111).^33−35^ Both the registry and the C–Cu distance (∼2.4–2.8 Å) suggest the presence of concurrent van der Waals and π-electron interactions. A similar effect takes place for the rotor in conformation C (C–Cu distance of ∼2.5–3.5 Å), while the stator adsorbs asymmetrically: One of the C-rings is flattening toward the surface (C–Cu distance ranging between 2.9 and 3.5 Å) and the second C-ring is pointing upward (bottom of Figure 2). Conformation B (center of Figure 2) shows an intermediate situation, with the stator mainly adsorbed via the S–Cu bond and the rotor distorted from the planar adsorption, due to the presence of the methyl group attached to the rotor.

This methyl group plays an important role in the adsorption: it points upward, that is, away from the surface, in conformation C, thus enabling the rotor to adsorb flatter and closer to the metal surface. Instead, in conformation B, the methyl is pointing downward and the rotor cannot adsorb flat on the surface, thus affecting the molecular stability on the substrate. Accordingly, C results as the most stable conformation, while A and B are ∼270 and ∼150 meV higher in energy, respectively (see below). Hence, conformations A and B, which are most abundant after sample preparation at 5 K (Figure 1c), are kinetically stabilized under the used adsorption conditions.

In addition to the adsorption energies of the entire molecule and its two parts with respect to the ground state structure in the gas phase, we also computed the same quantity with respect to the gas phase distorted structures optimized on the surface for each configuration. This was done with the aim to select the adsorption term only, that is without any contribution from distortions. This analysis (reported in the Supporting Information; Figures S2 and S3) shows that the majority (70%) of the adsorption energy of conformation A is caused by the stator–surface interaction that is overall stronger than for conformations B and C (0.38 and 0.21 eV higher in energy, respectively), indicating effective adsorption via the π-electrons. In configurations B and C, the adsorption energy is equally distributed between the two components, rotor and stator. C is ∼0.15 eV more stable than B, an energy contribution that again comes from increased π-interaction of the rather flat rotor with the surface in conformation C (see Figure 2).

A characteristic of Feringa-type molecular motors is their chirality, which is important for the uni-directionality of their rotation.^6,36^ It is the presence of a stereocenter in these molecules that upon an external stimulus, a change between two potential energy surfaces, a uni-directional rotary motion versus a random flapping process, is obtained. Accordingly, for all such chiral molecular motors, their enantiomers rotate in the opposite direction.^37^ The molecule studied here is not flat (as drawn for simplicity in the chemical structure, Figure 1a), but both rotor and stator are non-planar to each other, due to the twisted central alkene.^6^ This is valid both in solution^8,38^ and on the surface, as revealed by calculations (Figure 2).

**Figure 3:**
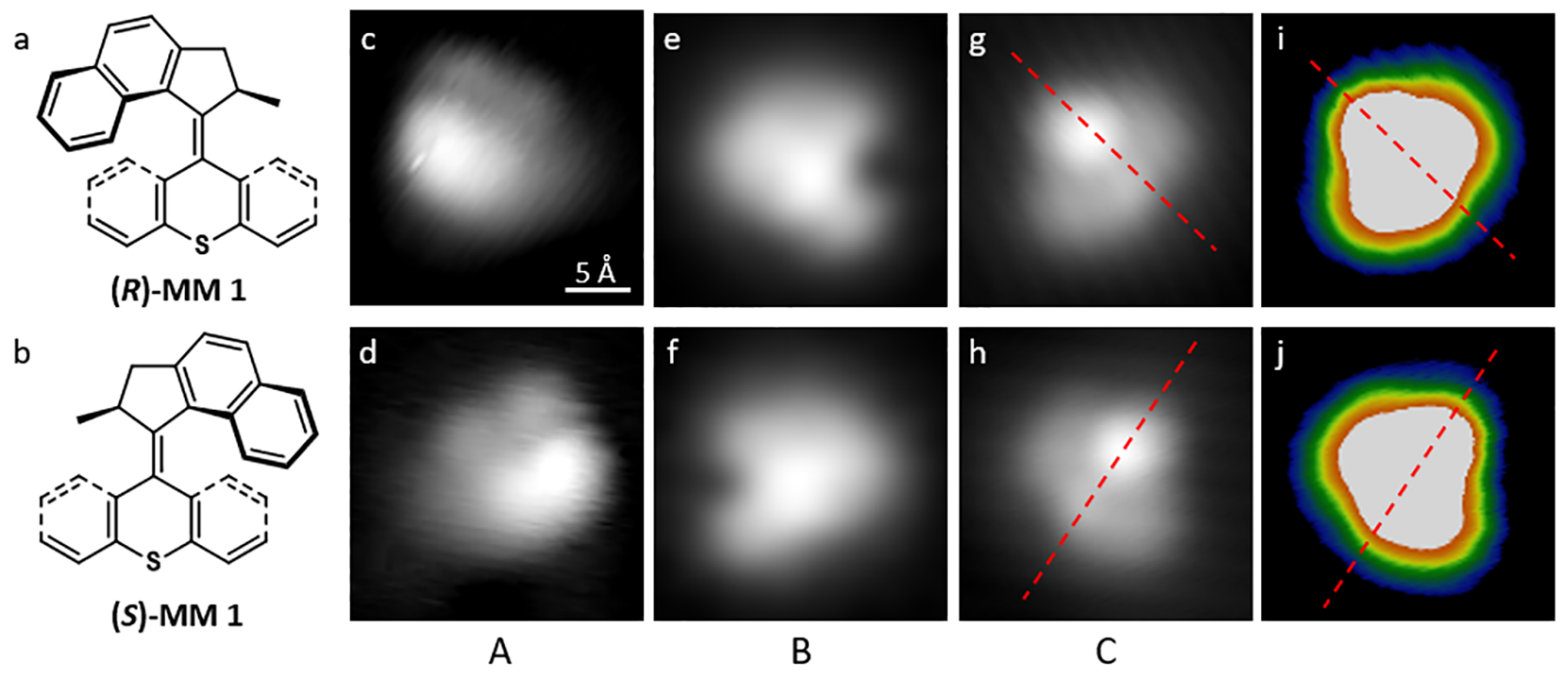
(a,b) Chemical structures of the two enantiomers (*R*) and (*S*) of **MM1**. (c–j) STM images [all 2.26 nm × 2.26 nm in size with 11 pA/300 mV (c,d), 10 pA/150 mV (e), 10 pA/100 mV (f) and 60 pA/−50 mV (g−j)] individual molecules in conformation A (c,d), B (e,f) and C (g,h) on the Cu(111) surface, which are assigned to the (*R*) (upper row) and (*S*) enantiomer (lower row) from their characteristic appearances. (i–j) Same STM images as in (g,h), but with a multicolor contrast to enhance the visibility of the chiral appearance with respect to the approximate symmetry axis of the molecular appearance (indicated by a dashed line).

It is known that the enantiomers of Feringa-motors maintain their chirality during the motor activity, which has been shown in solution where no racemization at the methyl-based stereogenic center takes place during any photochemical or thermal step.^6^ Synthesis of our molecules leads to a 1:1 mixture of the two enantiomers. Accordingly, and because sublimation temperatures are equal for both, the two enantiomers (Figure 3a,b) should arrive in about the same amount on the surface during deposition. This is confirmed by our experiments when studying the appearance of many molecules in view of their chirality. Figure 3c–h shows STM images of single molecules in the three conformations A, B, and C, and it is found that both enantiomers are present for each conformation. By comparing the upper with the lower row, it can be seen that in all three cases, the two enantiomers are mirror images of each other. While this is rather clear for conformations A and B, the difference is less evident for conformation C, and the corresponding STM images of the two enantiomers (Figure 3g,h) are therefore also plotted in multicolor contrast (Figure 3i,j). This enhances the visibility of its asymmetric shape with respect to the apparent symmetry conceivable on the basis of the regular gray scale STM image only (the approximate mirror axis, referring to the overall shape of the molecule, is plotted as dashed line).

**Figure 4:**
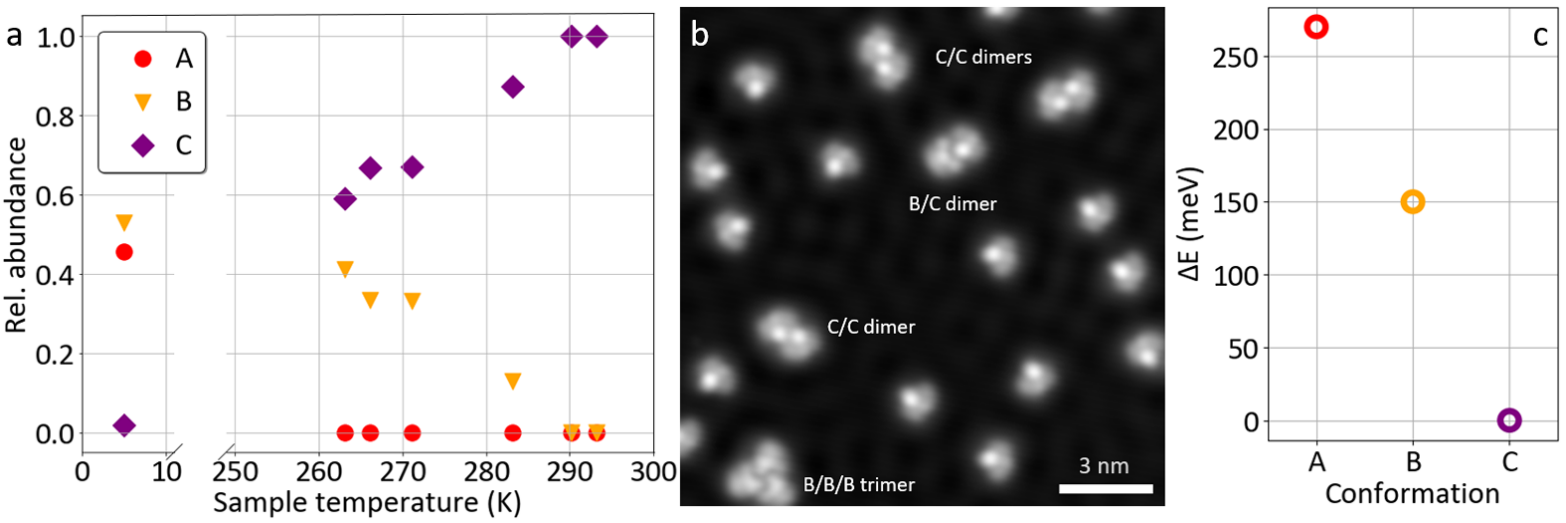
(a) Relative abundance of the three conformations (A, B, and C) on the surface for different sample temperatures during molecular deposition, as determined from the analysis of many STM images. (b) STM image (30 pA, −50 mV) of different individual molecules and small molecular assemblies of the motor molecules on Cu(111). Molecules were deposited onto the sample at a maximum temperature of 273 K. (c) Calculated total energies (see also Figure S2) of the three conformations on the Cu(111) surface, plotted as the energy difference Δ*E* with respect to conformation C (Δ*E* > 0 indicates a less preferred configuration).

The three conformations found on the surface are a result of the molecular flexibility in combination with the interaction between molecule and surface. A comparison of preparations with different sample temperatures during molecular deposition (Figure 4a) shows that their relative abundance depends strongly on the temperature: At 5 K sample temperature, all three conformations are present (see Figure 1c), C being very rare though. This is different at 263 K where A is completely absent, but the abundance of C has grown substantially. At higher temperatures (between 260 and 290 K) C becomes increasingly abundant (at the expense of B) until only C is found on the surface at room temperature. Hence, the initially dominant conformations A and B were thermally transformed into C. Note that post-preparation sample heating (i.e., molecule deposition at low temperatures and subsequent sample heating) leads to the same result. Thus, conformation C reflects the thermodynamic minimum within the parameter range that is explored here. Note that our experiments cannot directly resolve these thermally induced molecular transformations. A comparison of the different calculated structures (Figure 2) suggests that the conformational change from A to C reflects a helix inversion, while the molecule seems to undergo a 180° rotation of the rotor unit upon the transition from B to C.

The sample temperature affects not only the relative abundances of the three conformations, but also the assembly of the molecular motors as it influences the molecular mobility on the surface. After deposition at 268–283 K sample temperature, conformation A is absent and B can be found on the surface (as at low sample temperatures), but in contrast to lower temperatures, conformation B is never observed in isolated molecules. Instead, they are exclusively found assembled in dimers and particularly trimers (at the bottom of Figure 4b). This indicates their stabilization by kinetic effects, in contrast to the thermodynamic preference of conformation C. On the other hand, C prevails at this sample temperature and is often found for isolated molecules, but also in dimers (e.g., at the top of Figure 4b). The characteristic number of involved molecules for these two conformations, B and C, (three and two, respectively) indicates different interaction energies and thus stabilities in their packing. The majority of dimers consist of two molecules in conformation C. Additionally, combinations of different conformations within an assembly are also observed as for instance the dimer of conformations B and C in the center of Figure 4b.

**Figure 5:**
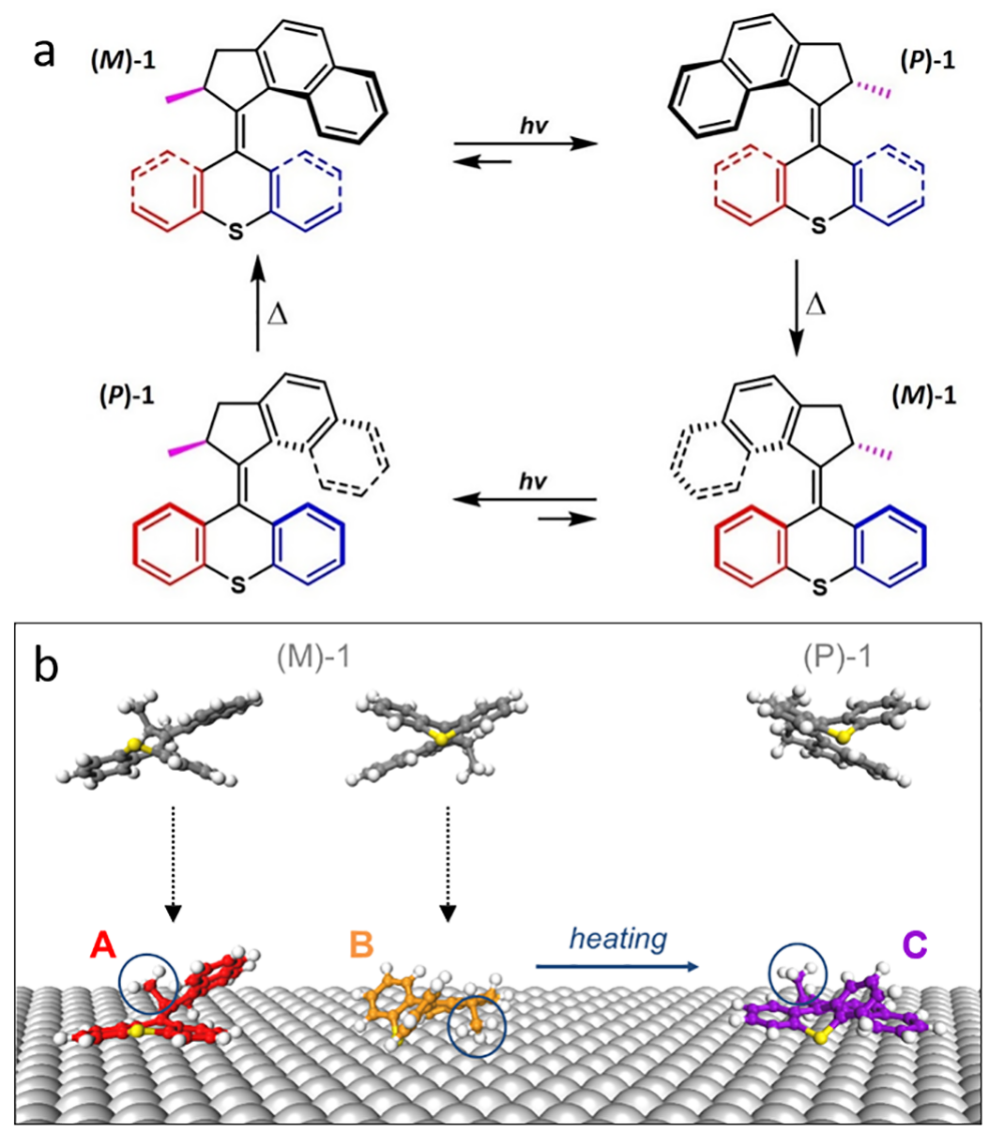
(a) Full 360° rotary cycle of the molecular motor. (b) Scheme of the molecular adsorption on the surface from the gas phase and the origin of the on-surface conformations A, B, and C (the important methyl group, see main text, is indicated by circles). Note that while all structures in (a,b) show the same enantiomer (*S*) for the sake of clarity, the same mechanisms are valid for the other enantiomer (*R*).

Our experimental observations of the molecular abundances agree well with the trend of calculated relative energies for the three conformations on the surface (Figure 4c), where C is the most stable and A is the highest in energy, that is the least stable conformation. Accordingly, higher sample temperatures (either during molecular deposition or during post-deposition heating) result in a pure composition of conformation C on the surface as observed (Figure 4a). However, the distribution at very low temperatures can only be understood from the atomic-scale picture of the calculated conformations (Figure 2), considering the different states of the molecular motor during its cycle. This cycle (shown in Figure 5a) consists of two energetically favored states (M)-1 and two unfavored states (P)-1. Their energy difference is a key property of the Feringa motor as it causes uni-directional thermal helix inversion (indicated in Figure 5a by the thermal step Δ).^8^

Considering the motor rotary cycle (Figure 5a) and the involved molecular conformations in solution, we can now analyze the adsorption behavior of the motor molecules. After synthesis, all molecules are in the (M)-1 state in both *S*- and *R*-enantiomers. During deposition under ultrahigh vacuum conditions, the molecules do not transform into the unfavored state (P)-1, because this process includes heating of the molecules to about 375 K. The molecules remain in state (M)-1 in the gas phase and land on the metal surface, adopting either conformation A or B (sketched at the left in Figure 5b). This is because A and B are the conformations that correspond in their internal structure to state (M)-1 of the gas phase. Importantly, the two conformations A and B differ mainly in their orientation, with the methyl group pointing either upwards (i.e. away from the surface; conformation A) or down towards the surface (conformation B; see Figure 2). In other words, A and B correspond to how the molecule (being (M)-1 in the gas phase) lands on the surface. The molecules can therefore statistically adsorb in both conformations with equal probability. This agrees very nicely with our experimental observation since the two conformations are found in almost the same abundance (45% in A vs 53% in B). Only the small remaining 2% is in conformation C, which reflects the fact that the corresponding conformation (P)-1 of the molecule is energetically unfavored in the gas phase (to emphasize this, no vertical arrow is drawn at the right of Figure 5b).

As the calculations show (Figure 4c), conformation A is much less stable than the others and it can therefore be expected to convert easily into other conformations as soon as sufficient thermal energy is provided. This is indeed what happens upon sample heating as conformations A and B are converted into conformation C (Figure 4a). In terms of the molecular motor rotary cycle, this means that state (M)-1 transforms on the surface to state (P)-1 (sketched at the right of Figure 5b), which is less stable in the gas phase (about 315 meV higher in energy than (M)-1 according to our DFT calculations), but the more stable state upon surface adsorption (about 270 and 150 meV lower in energy than A and B, respectively, Figure 4c). Hence, the energetic landscape is distorted by the surface and sample heating causes an “inverted” helix rotation as the system relaxes into the preferred state (P)-1, which corresponds to conformation C. This effect is of great interest for future studies of the molecular function, because the motor activity and its uni-directional rotation are based on the energetic difference between states (M)-1 and (P)-1 and the corresponding helix inversion upon thermally induced relaxation. Accordingly, it can be expected that the molecular motor rotary cycle might change after adsorption on the surface. Investigations of the molecular dynamics on Cu(111) are currently in progress.

## Conclusions

Molecular motors were adsorbed on a Cu(111) surface and studied at the single-molecule level to determine molecular conformations on the surface. We find that the presence of the metal surface strongly distorts the potential energy landscape of the molecular motor states, inverting the stability of the characteristic states of the motor molecule. Specifically, the (M)-1 state, which is the most stable configuration in solution, becomes less stable for the benefit of the (in the gas phase unfavored) (P)-1 state. We anticipate that this should have a strong effect on the thermal relaxation step within the motor rotary cycle and might also affect the starting point of the cycle, which is always assumed to be the (M)-1 state (top left in Figure 5a), but appears to be changed [to (P)-1] on the surface.

## Supporting Information Available

Adsorption energies and electronic properties from first principles DFT simulations and post-processing of simulated STM images (PDF)
