# Supplementary material for: Inverted Conformation Stability of a Motor Molecule on a Metal Surface
Source: J Phys Chem C Nanomater Interfaces. 2022 May 18;126(21):9034–40. doi: 10.1021/acs.jpcc.2c00406 (PMC9169611; doi:10.1021/acs.jpcc.2c00406)
Supplement: Supplementary file 1 — jp2c00406_si_001.pdf [file jp2c00406_si_001.pdf]

Inverted Conformation Stability of a Motor Molecule on a Metal Surface

Monika Schied,[1] Deborah Prezzi,[2*] Dongdong Liu,[3] Peter Jacobson,[1†] Stefano Corni,[2,4] James M. Tour,[3*] and Leonhard Grill[1*]

1) Department of Physical Chemistry, University of Graz, Heinrichstraße 28, 8010 Graz, Austria

2) Nanoscience Institute of the National Research Council (CNR-NANO), via G. Campi 213/a, 41125 Modena, Italy

3) Departments of Chemistry and Materials Science and NanoEngineering, the Smalley Institute for Nanoscale Science and Technology, the Welch Institute for Advanced Materials, Rice University, Houston, Texas 77005, United States

4) Dipartimento di Scienze Chimiche, Università di Padova, I-35131 Padova, Italy

* Corresponding authors: deborah.prezzi@nano.cnr.it (D.P.), tour@rice.edu (J.M.T.) and leonhard.grill@uni-graz.at (L.G.)

◊ Present address: School of Mathematics and Physics, The University of Queensland, Brisbane, Queensland 4072, Australia

Contents

Post-processing of simulated STM images S2
Adsorption energies from first principles DFT simulations S2
Electronic properties from first principles DFT simulations S3
References S4

Post-processing of simulated STM images

The simulated STM images were obtained using the Tersoff-Hamann approximation. This yields images with much more details than what can be resolved in the actual measurements, due to the limited sharpness of the STM tip. For better comparison between simulation and experiments, the simulated images are therefore artificially blurred, using a Gaussian filter with a radius of 15 px using the software FIJI[1]. A comparison of images is shown in Fig. S1.

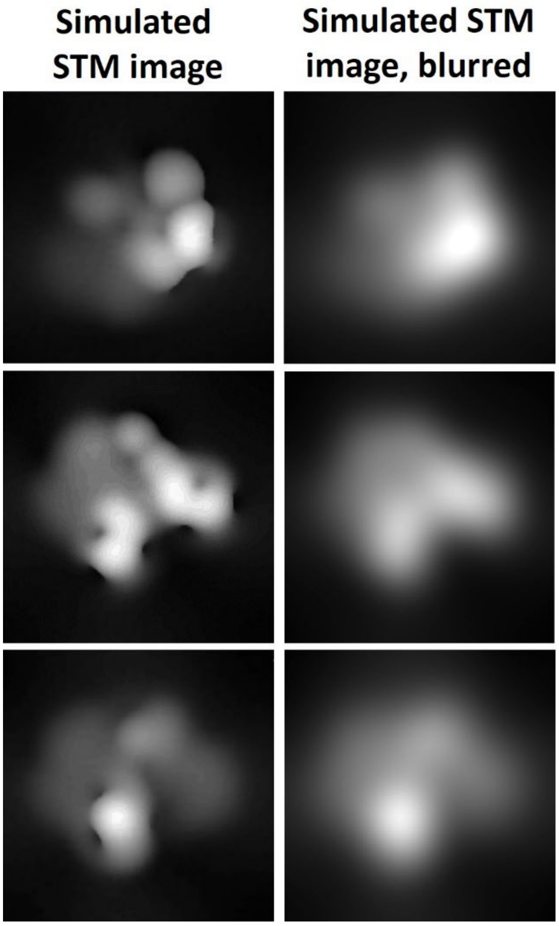

Figure S1. *Comparison of original simulated STM images and the same images that were blurred artificially for a better comparison with experimental images.*

Adsorption energies from first principles DFT simulations

To characterize the different configurations, i.e. A, B and C (see main text), of the molecular motor **MM1** on Cu(111), their adsorption energies ($E_{ads,X}$) were calculated from the following expression:

$$E_{ads,X} = -\left[\, E_{MM1(X)/Cu(111)} - \left(E_{MM1} + E_{Cu(111)} \right) \right]$$

E_{MM1}, $E_{Cu(111)}$ and $E_{MM1(X)/Cu(111)}$ represent the DFT total energies of the molecule in gas phase, the optimized Cu(111) slab alone and the Cu(111) slab covered with the molecule in configuration X (X = A, B and C), respectively. Note that the energy E_{MM1} always refers to the (M)-1 state of the molecule (in the gas phase). To gain insight into the driving force of adsorption, the adsorption energies were also computed for the two separate components of **MM1**, i.e. stator and rotor (see Fig.1a in the main text), in all configurations. This was done by taking the optimized structure of **MM1** (either isolated

or on the Cu(111) surface), removing the rotor (stator) portion, saturating the C radical with H atoms and computing the self-consistent total energy of the resulting structures without any ionic optimization. The results are reported in the top panel of Fig. S2. For configurations B and C, there are three bars, corresponding (from left to right) to the entire molecule (**MM1**), the stator and the rotor contributions. For configuration A, only the entire molecule and the stator contribution are shown, since the rotor points away from the surface at a distance larger than 3.6 Å and the direct interaction with the substrate is less relevant.

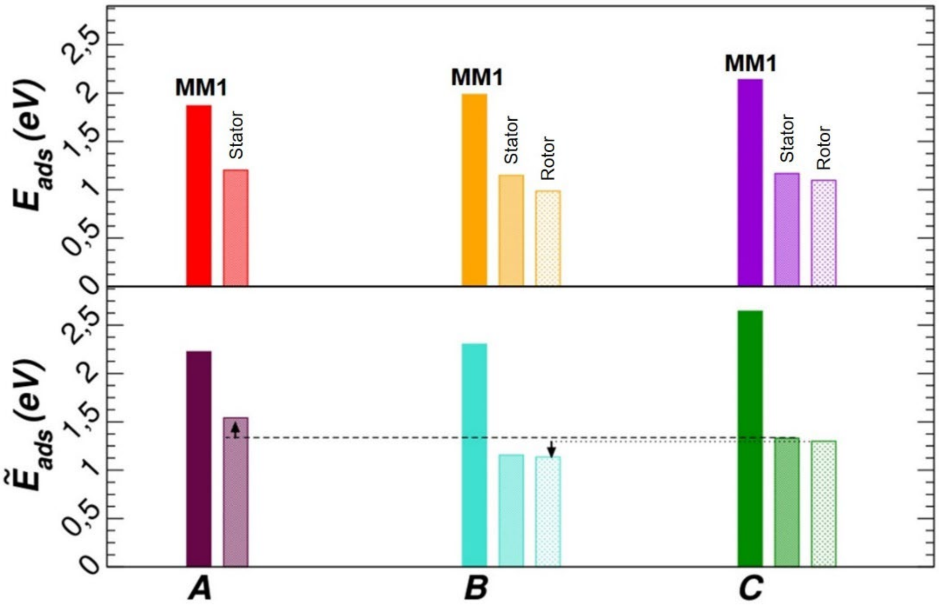

Figure S2. *Adsorption energies (E_{ads}, eV) computed from the DFT total energies of each investigated configuration, i.e. X = A, B, C. Top (bottom) panel reports E_{ads} ($\tilde{E}_{ads}$) computed with respect to the isolated (distorted) molecule, as defined in the text. For each configuration, E_{ads} ($\tilde{E}_{ads}$) is plotted (from left to right) for the entire molecule (MM1), the stator and the rotor parts (only molecule and stator for A).*

As defined above, the adsorption energy of the entire molecule is computed with respect to the total energy of the isolated molecule optimized in gas phase (the same is valid for calculations of rotor or stator portions). However, this value inherently contains both the energetic contribution related to the adsorption process and the contribution coming from potential structural distortions arising upon adsorption. Since molecular distortions on Cu(111) are significant, an additional adsorption energy term $\tilde{E}_{ads,X}$ was computed by using the total energy $\tilde{E}_{MM1,X}$ of the isolated distorted structures (coming from on-surface optimization) for each configuration X, in order to single out the adsorption term only, i.e. without any contribution caused by distortions. This further analysis is reported in the bottom panel of Figure S2.

Electronic properties from first principles DFT simulations

For each investigated configuration, the electronic properties were computed within the DFT framework. Figure S3 displays for each adsorbed configuration the molecular projected density of states (pDOS), that is, the density of states projected onto the atomic orbitals of the atoms constituting the molecule (colored solid lines). This allows one to highlight the HOMO-LUMO gap of the adsorbed molecule in each configuration, to be compared with that of the molecule in gas-phase

(grey area). Noticeably, the HOMO-LUMO gap on the substrate shows a shrinkage for all three configurations, which can be mainly attributed to the molecule distortions induced upon absorption. This is evident by comparing the pDOS of each molecular configuration to the DOS of the corresponding isolated distorted molecule (dashed colored lines with filled areas), with the HOMO-LUMO gap of B being the least affected one since B is the least distorted configuration.

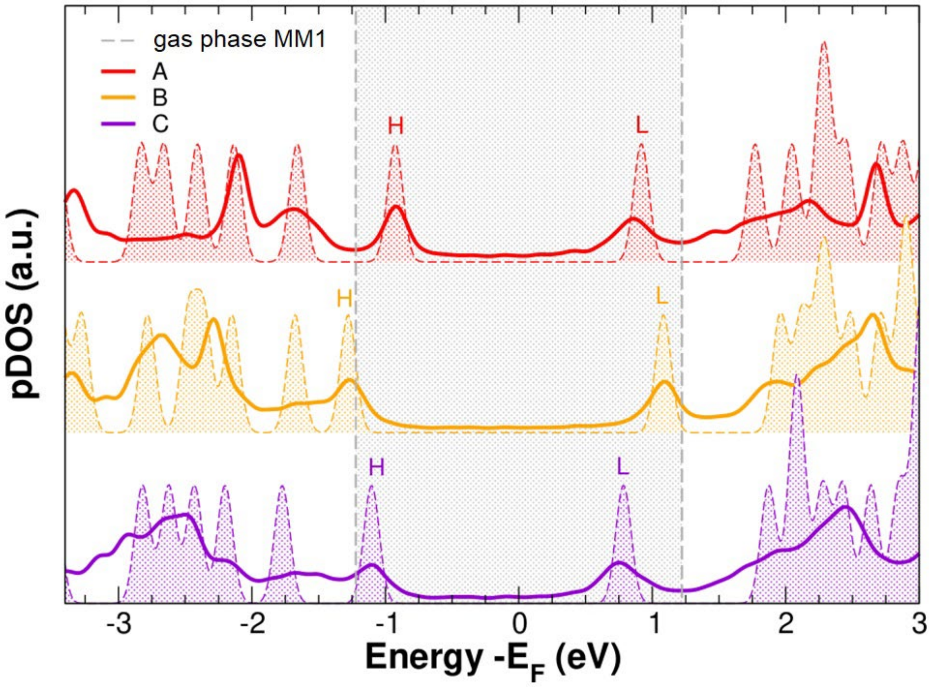

Figure S3. *Projected density of states (pDOS) for each adsorbed configuration, i.e. A (red), B (orange), C (violet). The pDOS of each molecular configuration on the substrate (coloured solid lines, according to the colour code defined above) is plotted together with the DOS of the corresponding distorted structure computed in the gas phase. The grey area indicates the HOMO-LUMO gap of the isolated **MM1** molecule, centred around the Fermi level. The pDOS for the three molecular configurations are displaced vertically for clarity*

References

1. Schindelin, J.; Arganda-Carreras, I.; Frise, E.; Kaynig, V.; Longair, M.; Pietzsch, T.; Preibisch, S.; Rueden, C.; Saalfeld, S.; Schmid, B.; Tinevez, J.-Y.; White, D. J.; Hartenstein, V.; Eliceiri, K.; Tomancak, P.; Cardona, A., Fiji: an open-source platform for biological-image analysis. *Nat. Meth.* **2012,** *9*, 676-682.
